# Isoquercitrin activates the AMP–activated protein kinase (AMPK) signal pathway in rat H4IIE cells

**DOI:** 10.1186/1472-6882-14-42

**Published:** 2014-02-03

**Authors:** Jingxin Zhou, Hisae Yoshitomi, Tonghua Liu, Boxin Zhou, Wen Sun, Lingling Qin, Xiangyu Guo, Liansha Huang, Lili Wu, Ming Gao

**Affiliations:** 1 Beijing University of Chinese Medicine, 11 North 3rd-ring East Road, Beijing, Chaoyang District 100029, People’s Republic of China; 2 School of Pharmaceutical Sciences, Mukogawa Women’s University, 11-68 Koshien Kyuban-cho, Nishinomiya, Hyogo 663-8179, Japan; 3 Sanshui Hospital affiliated to Guangdong Medical college, 16 Guanghai West Road, Foshan, Guangdong 528000, People’s Republic of China; 4 Shenzhen TCM Hospital, 1 Fuhua Road, Shenzhen, Guangdong, Futian District 518000, People’s Republic of China

**Keywords:** Isoquercitrin, H4IIE cells, AMPK, AdipoR1

## Abstract

**Background:**

Isoquercitrin, a flavonoid compound that is widely distributed in medicinal and dietary plants, possesses many biological activities, including inhibition of adipocyte differentiation. In this study, we investigated the effect of isoquercitrin on lipid accumulation and its molecular mechanisms in rat hepatoma H4IIE cells.

**Methods:**

To investigate the effect of isoquercitrin on lipid accumulation, H4IIE cells were induced by FFA and the total lipid levels were detected by Oil Red O staining. Furthermore, The protein levels of AMPK and acetyl-CoA carboxylase (ACC), the gene expressions of transcriptional factor, lipogenic genes, and adiponectin receptor 1 (AdipoR1) were analyzed by Western blotting and quantitative real-time PCR. To further confirm the pathway of isoquercitrin-mediated hepatic lipid metabolism, H4IIE cells were treated with an AMPK inhibitor and AdipoR1 siRNA.

**Results:**

Isoquercitrin significantly enhances AMPK phosphorylation, downregulates sterol regulatory element binding protein transcription factor 1 (SREBP-1) and fatty acid synthase (FAS) gene expressions. Pretreatment with AMPK inhibitor, significantly decreased the AMPK phosphorylation and increased FAS expression stimulated by isoquercitrin. Isoquercitrin might also upregulate the expression of AdipoR1 dose-dependently via AMPK in the presence of an AMPK inhibitor and AdipoR1 siRNA.

**Conclusions:**

Isoquercitrin appears to regulate AMPK activation, thereby enhancing AdipoR1 expression, suppressing SREBP-1 and FAS expressions, and resulting in the regulation of lipid accumulation. These results suggest that isoquercitrin is a novel dietary compound that can be potentially be used to prevent lipid metabolic disorder and nonalcoholic fatty liver disease.

## Background

Adenosine monophosphate-activated protein kinase (AMPK) has been recognized as a master regulator of hepatic metabolism. Its activation results in an increase in fatty acid oxidation and suppression of fatty acid synthesis via inactivation of acetyl-CoA carboxylase (ACC). It inhibits the lipogenic gene expression of fatty acid synthase (FAS) by decreasing the action of transcription factors [1]. Sterol regulatory element binding protein 1 (SREBP-1) is the most important transcription factor regulating the expression of lipogenic enzymes, including ACC and FAS. Many studies have suggested that increased levels of SREBP-1 are closely associated with fatty liver in obese animal models [2]. Meanwhile, a previous report has shown that SREBP-1 is mediated by AMPK [3]. Therefore, AMPK plays a key role in fatty acid metabolism in the liver and in whole body lipid metabolism.

Lipid metabolic disorder refers to a disruption in lipid metabolism in which there are excessive serum levels of lipids and lipoprotein lipids. This disorder has escalated into a major worldwide public health problem, and it is a critical risk factor for metabolic syndromes, such as insulin resistance, type 2 diabetes mellitus, and cardiovascular diseases [4]. Nonalcoholic fatty liver disease (NAFLD) develops when a large amount of lipid is deposited in hepatocytes, and it is tightly associated with lipid metabolic disorder [5]. Untreated NAFLD may progress to more serious diseases, such as liver cirrhosis and hepatocellular carcinoma, and, consequently, influence other body systems to cause atherosclerosis, type 2 diabetes, and hypertension [6]. Therefore, it is essential to identify ways to prevent fatty liver. Treatment of fatty liver focuses on inhibition of excessive free fatty acids (FFAs), either by reducing hepatic lipogenesis or by increasing oxidation or export.

Recently, adiponectin receptors (AdipoRs) and AMPK signaling have been recognized as critical regulators of hepatic lipid metabolism to treat fatty liver. AdipoRs and AMPK signaling suppress fatty acid synthesis by inactivating ACC and FAS and stimulate fatty acid oxidation. SREBP-1, a key lipogenic transcription factor, plays a major role in regulating hepatic lipid metabolism, including lipolysis and fatty acid synthesis [7]. Inhibition of SREBP-1 expression by activating AMPK reduces lipid accumulation in hepatocytes to ameliorate fatty liver. Currently, many pharmaceutical chemicals for the treatment of fatty liver, such as insulin sensitizers and lipid-lowering drugs, have the risk of side effects. In contrast, natural dietary compounds, which can ameliorate steatosis, may have fewer risks. These natural compounds also play an important role in improving the quality of life and maintaining health. Therefore, the discovery of food components that can prevent and treat NAFLD is of interest and value.

Isoquercitrin (quercetin3-O-b-D-glucopyranoside) is a natural flavonoid glucoside that is distributed in medicinal and dietary plants, such as vegetables, herbs, and flowers [8]. Isoquercitrin has been found to have a wide range of biological properties, such as anti-inflammatory effects [9]; antioxidant activity, including decreasing ROS levels and reducing lipid peroxidation both *in vivo* and *in vitro*[10]; neuroprotection; and promotion of neurite elongation [11]. In particular, a recent study reported that isoquercitrin inhibits adipocyte differentiation of 3 T3-L1 cells via activation of Wnt/β-catenin signaling [12]. However, it is not clear whether isoquercitrin mediates AMPK signaling to result in amelioration of lipid metabolism in hepatocytes.

Recently, many studies in cell lines and animal models have examined the ability of food and dietary plant components to prevent fatty liver and hepatic steatosis by mediating hepatic lipid accumulation. Research has focused on luteolin [13] and anthocyanins [14]. Additionally, studies have indicated that isoquercitrin may possess the capability to reduce lipid peroxidation and inhibit adipocyte differentiation. However, it has not been clarified *in vitro* whether isoquercitrin regulates the hepatic AMPK pathway, which plays a critical role in mediating lipid metabolism and fatty accumulation. In the present study, we investigated the effects of isoquercitrin on the management of the AMPK signaling pathway in rat hepatoma H4IIE cells.

## Methods

### Reagents

Isoquercitrin was purchased from the National Institutes for Food and Drug Control (Beijing, China). Dulbecco’s modified Eagle’s medium (DMEM) and antibiotic solution was obtained from Nacalai Tesque (Kyoto, Japan). AMPK inhibitor, compound C was pucharsed from Santa Cruz Biotechnology, Inc. (Texas, USA), and 5-aminoimidazole-4-carboxamide 1-ribofuranoside (AICAR) was purchased from Cell Signaling Technology, Inc. (Beverly, USA).

### Cell culture

Rat hepatoma (H4IIE) cells, which were purchased from The Japanese Collection of Research Bioresources Cell Bank (Osaka, Japan), were cultured in Dulbecco’s Modified Eagle Medium (DMEM) supplemented with 10% fetal bovine serum (FBS) and 1% streptomycin/penicillin (Nacalai Tesque, Kyoto, Japan) at 37°C in a 5% CO_2_ humidified atmosphere. Cells at 70–80% confluence were exposed to various concentrations of isoquercitrin (50, 100, or 200 μM) or to 1 μL dimethyl sulfoxide (DMSO) as a control for the indicated times. All of the cells were incubated at 37°C in a 5% CO_2_ humidified atmosphere.

### MTT assay

H4IIE cells were plated at 5000 cells/well in 96-well tissue culture plates. The indicated concentrations of isoquercitrin (0, 50, 100, and 200 μM) dissolved in DMSO were added and incubated for 72 h. MTT reagent (0.01 ml, 5 mg/ml) was added and incubated in each well for 3 h. The MTT medium was replaced with 0.1 ml DMSO, and absorbance was read at 570 nm. Cell viability was calculated by the absorbance of isoquercitrin-treated cells compared to control cells.

### Oil Red O staining and measurement of lipid accumulation

Oil Red O powder (150 mg) was dissolved in 50 ml of isopropanol to form a stock solution, which was diluted with distilled water to 60% of the stock concentration. After filtration, the supernatant was stored at room temperature. Subconfluent H4IIE cells were cultured with the control group (DMSO) and the given concentrations of isoquercitrin (50, 100, or 200 μM) in DMEM for 24 h. Next, the cells were treated with 0.1 mM FFA for another 24 h to produce lipid accumulation. The cell layers were washed with phosphate-buffered saline (PBS), fixed in 10% formalin for 10 min, stained with Oil Red O dye solution for 20 min at room temperature, washed with 60% isopropanol to remove unbound dye, washed with PBS, and then photographed under a microscope. Subsequently, the Oil Red O stain was dissolved in isopropanol, and lipid accumulation was measured by absorbance at 540 nm.

### Western blotting

Confluent cells with or without isoquercitrin were prepared with 100 μl of ice-cold homogenization buffer containing 50 mM Tris–HCl (pH 7.4), 100 mM NaCl, 1% Nonidet-P40, 0.25% sodium deoxycholate, 0.1% sodium dodecyl sulfate (SDS), 1 mM ethylenediaminetetraacetic acid (EDTA), 50 mM NaF, 2 mM Na_3_VO_4_, 30 mM sodium pyrophosphate, 2 mM phenylmethanesulfonylfluoride (PMSF), 1 mM benzamidine, 0.02 g/mL trypsin inhibitor, 0.02 g/mL leupeptin, and 0.02 g/mL aprotinin. After incubation on ice for 30 min, the lysates were centrifuged at 12,000 rpm for 10 min, and the supernatants were isolated. Proteins were extracted by boiling in 0.5 mmol/l Tris–HCl, pH 6.8, 20% glycerol, 10% SDS, 0.1% bromophenol blue, and 10% 2-mercaptethanol. Equal amounts of protein (20 μg) were electrophoresed on a 7.5–12.5% sodium dodecyl sulfate polyacrylamide gel electrophoresis (SDS-PAGE) gel at 100 V for 2 h, and transferred onto polyvinylidene fluoride (PVDF) membranes (Amersham Life Science, Inc., Buckinghamshire, UK) at 100 mA for 2 h. The membranes were blocked in Blocking One-P and Blocking One (Nacalai Tesque, Kyoto, Japan) for 30 min. After blocking, the membranes were incubated overnight at 4°C with the following rabbit primary antibodies (at a dilution of 1:1000 in antibody solution 1 [Toyobo, Osaka, Japan]): p-AMPKα Thr172, t-AMPKα, p-ACC Ser79, or t-ACC (Cell Signaling Technology, Beverly, USA). The membranes were then washed with Tris-buffered saline and Tween 20 (TBST) and incubated with horseradish peroxidase-linked anti-rabbit secondary antibodies (1:10,000) for 1 h at room temperature. Detection was achieved using Ez Capture ST (ATTO, Tokyo, Japan) with Chemi-Lumi One Super (Nacalai Tesque, Kyoto, Japan). Bands were quantified and scanned using NIH Image. β-Actin was used as an internal control.

### Quantitative real-time PCR

Total RNA was isolated using Sepasol-RNA I Super G (Nacalai Tesque, Kyoto, Japan). Real-time PCR kits were from TOYOBO (Tokyo, Japan). RNA (1 μg) from each sample was reverse-transcribed to cDNA using the ReverTra Ace qPCR RT Kit, according to the manufacturer’s instructions (Toyobo). THUNDERBIRD SYBR qPCR Mix was used for quantitative real-time RT-PCR analysis of each gene’s expression. The following primers (Invitrogen, California, USA) were used: AdipoR1 forward, TGAGGTACCA GCCAGATGTC; AdipoR1 reverse, CGTGTCCGCT TCTCTGTTAC; FAS forward, GGAACTGAAC GGCATTACTC G; FAS reverse, CATGCCGTTA TCAACTTGTC C; SREBP-1 forward, CCACCCTGTA GGTCACCGTT T; SPEBP-1 reverse, GTGGGTATAA GCGTTCAGCT GC; β-actin forward, GGGAAATCGT GCGTGACATT; and β-actin reverse, GCGGCAGTGG CCATCTC. The amplification was performed as follows: 95°C for 1 min followed by 40 cycles of 95°C for 15 s, 60°C for 32 s, 95°C for 15 s, 60°C for 20 s, and 95°C for 15 s with a real-time PCR system (ABI Prism 7000). The data were normalized to β-actin.

### Knockdown with small interfering RNA (siRNA)

H4IIE cells were seeded in a six-well plate at 2 × 10^5^ cells per well in 2 ml of antibiotic-free normal growth medium supplemented with FBS. Subconfluent cells were transfected with AdipoR1 siRNA or control siRNA (Santa Cruz Biotechnology) in 1 ml Opti-MEM (GIBCO, USA) containing a transfection reagent mixture of Reagent A and B (Santa Cruz Biotechnology, USA) for 7 h. Then, 1 ml of 2 × normal growth medium was added. After a 24-h incubation, the normal growth medium was used for another 48 h according to the manufacturer’s instructions. The transfection efficiency was assessed by qRT-PCR, followed by treatment with isoquercitrin.

### Statistical analysis

Data for individual groups were presented as the mean ± standard error of the mean (SEM) and were statistically analyzed using Dunnett or Tukey test. Values between two groups were analyzed by Student’s *t*-test. Differences were considered significant for *P* < 0.05.

## Results

### Isoquercitrin inhibits lipid accumulation in FFA-induced H4IIE cells

**Figure 1:**
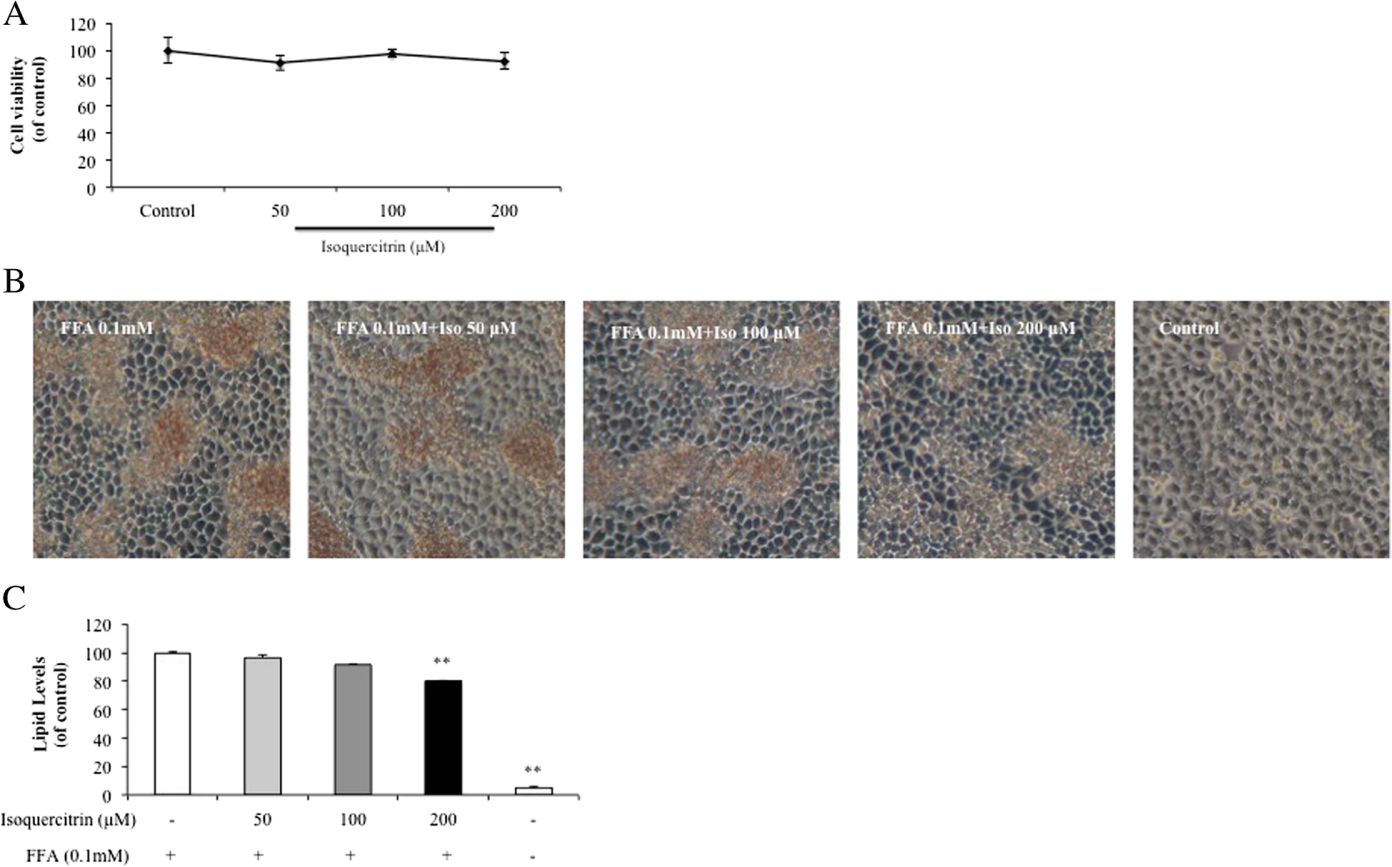
**Effects of isoquercitrin on cell survival and lipid accumulation in H4IIE cells with FFA. (A)** H4IIE cells were exposed to various doses of isoquercitrin, and cell viability was measured by MTT tests. Values are expressed as the percentage of the control, which was taken as 100%, and shown as the mean ± SEM (n = 3). **(B)** and **(C)** Cells were cultured in the absence or presence of isoquercitrin (50, 100, and 200 μM) for 24 h, followed by stimulation with or without FFA (0.1 mM) for another 24 h. **(B)** Intracellular oil droplets were visualized by Oil Red O staining under a microscope at 200 × original magnification. **(C)** Quantitative analysis of lipid deposition was measured by optical density (OD) values at 540 nm after staining. Values are presented as a dose-dependent decrease and expressed as mean ± SEM (n = 3). **P < 0.01 compared to FFA-treated control group.

Cells were cultured with different concentrations of isoquercitrin (50, 100, and 200 μM) for 72 h, and cell viability was determined by the MTT assay. As expected, isoquercitrin did not show significant cytotoxic effects (Figure 1A). Thereafter, all indicated doses of isoquercitrin were chosen to investigate the effect of isoquercitrin on lipid accumulation in H4IIE cells. Subconfluent H4IIE cells were incubated in medium containing the indicated concentrations of isoquercitrin for 24 h, followed by adding 0.1 mM FFA for another 24 h. The total lipid levels were detected by Oil Red O staining. As shown in Figure 1B and C, isoquercitrin treatment significantly prevented cellular lipid droplet accumulation in H4IIE cells in a dose-dependent manner.

### Isoquercitrin increases phosphorylation of AMPKα in H4IIE cells

**Figure 2:**
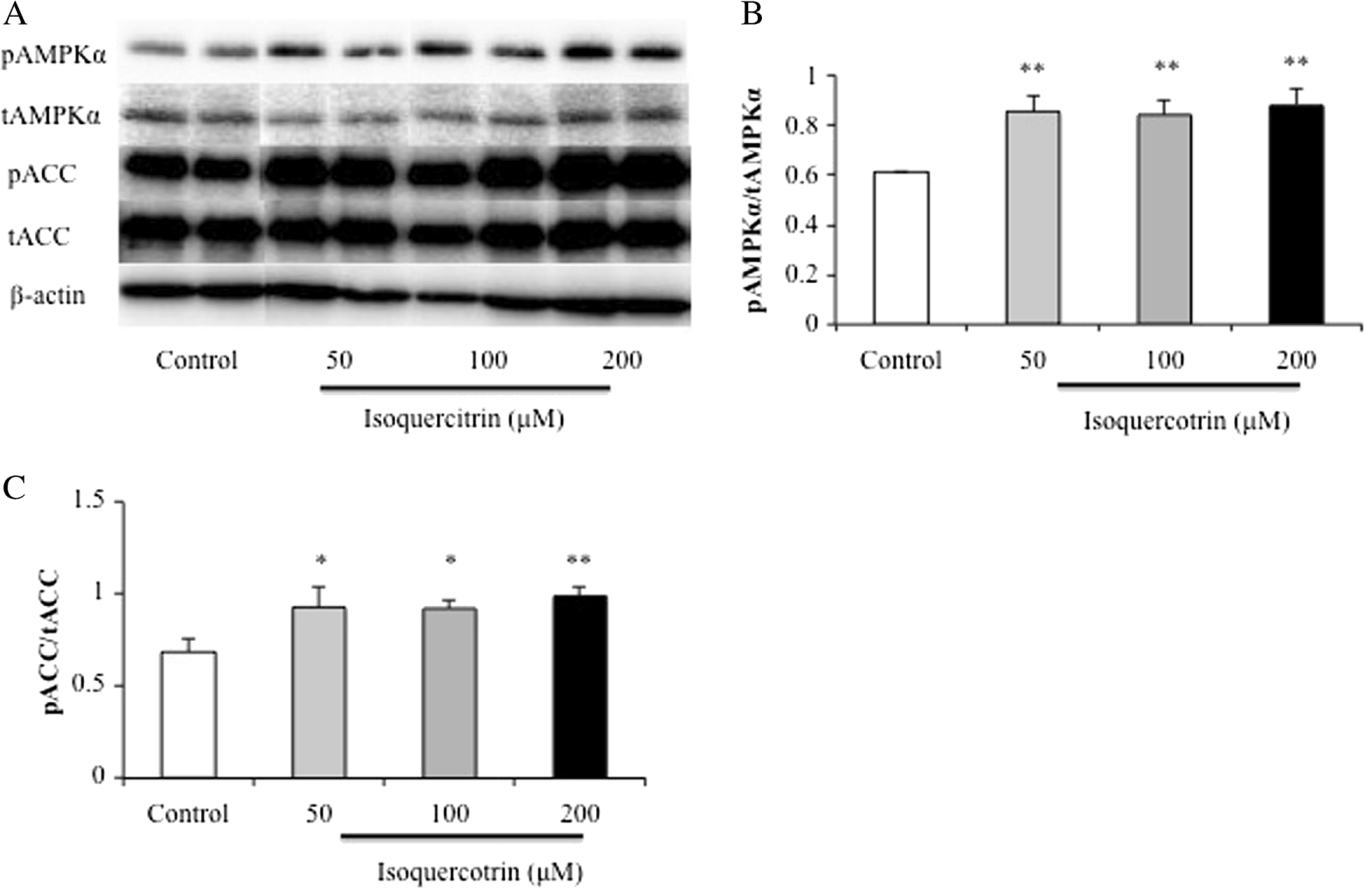
**Isoquercitrin stimulates phosphorylation of AMPK and ACC in H4IIE cells.** Cells were incubated with various concentrations of isoquercitrin or control for 12 h. **(A)** Total cell lysates were subjected to Western blot analysis with antibodies for phosphorylated AMPKα and ACC, total AMPKα, and ACC in H4IIE cells. **(B)** and **(C)** Values are shown as mean ± SEM (n = 3). *P < 0.05; **P < 0.01 compared to control group.

To assess the effect of isoquercitrin on phosphorylation of AMPKα that was the possible mechanism responsible for the lipid accumulation, H4IIE cells were treated with isoquercitrin (50, 100, and 200 μM) for 12 h. As Thr172 phosphorylation of AMPKα is an essential marker of AMPK activity, and ACC is its primary downstream targeting enzyme. The phospho-Thr172 antibody of AMPKα and the phospho-Ser79 antibody of ACC were examined by immunoblotting to assess AMPK activation. Isoquercitrin significantly increased the level of pAMPKα over the untreated control, but the expression of endogenous total AMPKα protein showed almost no change (Figure 2A). Moreover, isoquercitrin stimulated phosphorylation of ACC (Ser79), whereas the overall ACC protein expression did not change (Figure 2A). This result indicated that isoquercitrin enhanced the phosphorylation of AMPK and ACC (Figure 2B and C).

### Isoquercitrin downregulates SREBP-1 and FAS mRNA expressions in H4IIE cells

**Figure 3:**
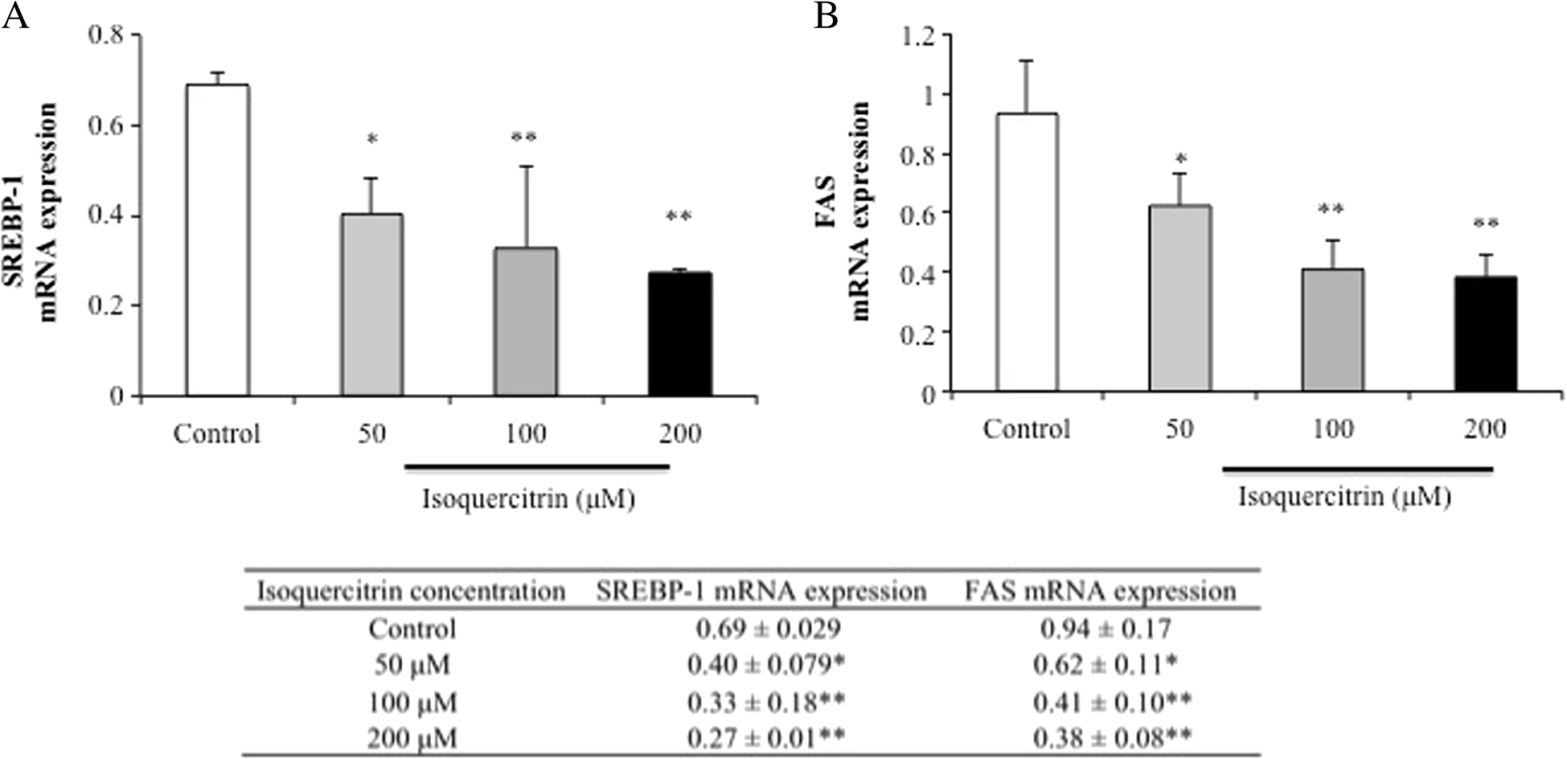
**Isoquercitrin decreases the mRNA expression levels of SREBP-1 and FAS in H4IIE cells.** Cells were treated with the indicated concentrations of isoquercitrin for 12 h. The mRNA expression levels of SREBP-1 **(A)** and FAS **(B)** were assessed by quantitative real-time PCR. Results are expressed as means ± SEM (n = 3) normalized to β-actin mRNA expression. *P < 0.05; **P < 0.01 compared to control group.

Many reports have shown that AMPK decreases the actions of the transcriptional factor SREBP-1 and suppresses the expression of lipogenic genes, including FAS [15]. Because isoquercitrin enhanced phosphorylation of AMPK and stimulated ACC phosphorylation, the effects of isoquercitrin on the gene expression of SREBP-1 and FAS in H4IIE cells were evaluated by real-time PCR analysis. H4IIE cells treated with isoquercitrin showed significantly decreased SREBP-1 and FAS mRNA expressions (Figure 3A and B).

### AMPK pathway regulates isoquercitrin-induced reduction of lipid levels in H4IIE cells

**Figure 4:**
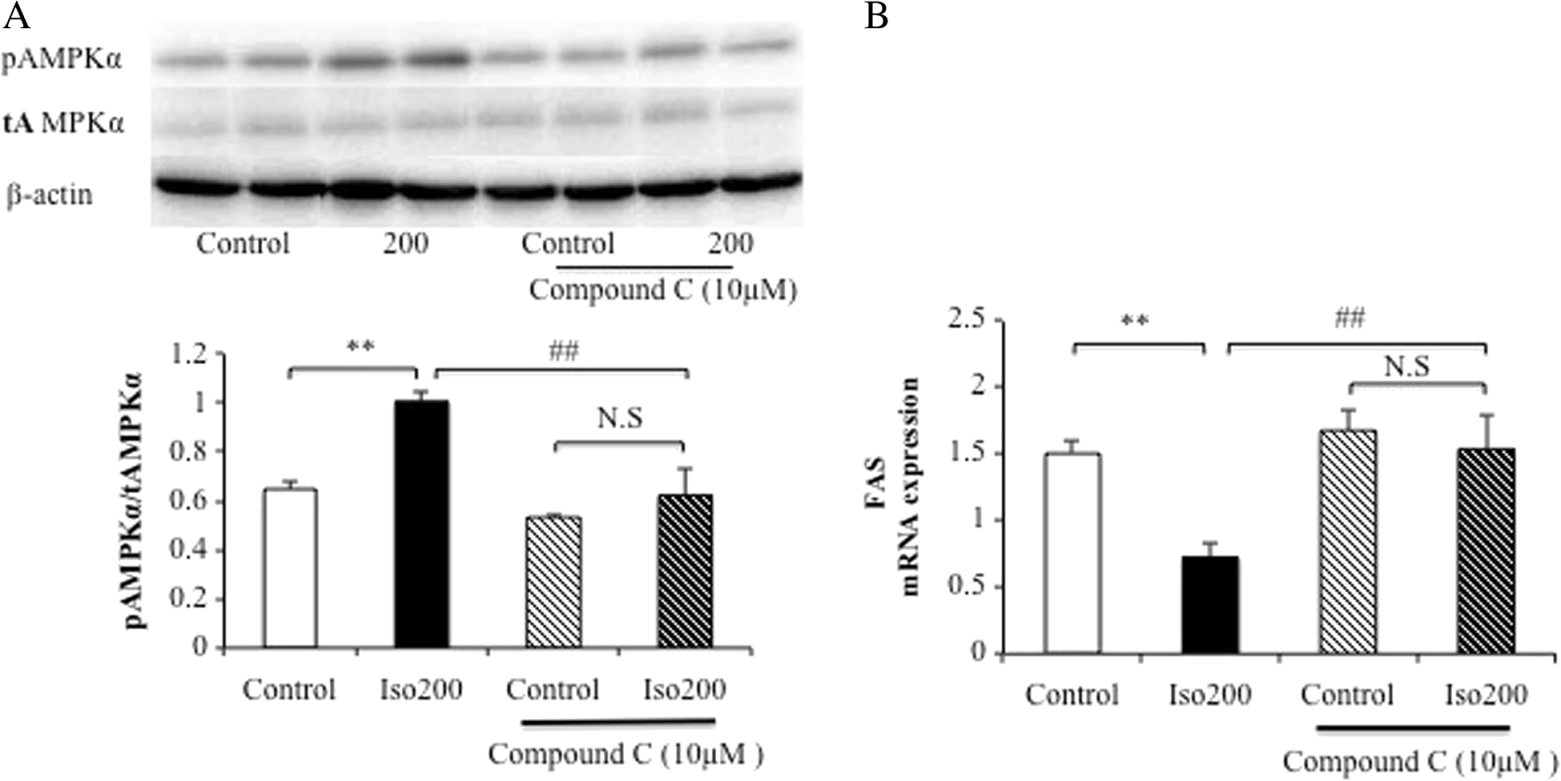
**AMPK inhibitor attenuates isoquercitrin-mediated FAS mRNA expression in H4IIE cells.** H4IIE cells were incubated with isoquercitrin (200 μM) in the absence or presence of compound C (10 μM) pretreatment for 1 h. **(A)** Isoquercitrin-induced AMPK activation was suppressed by compound C. **(B)** The mRNA expression level of FAS was analyzed as indicated. Values are expressed as mean ± SEM (n = 3). **P < 0.01 between control and isoquercitrin without compound C, ##*P* < 0.01 between isoquercitrin with or without compound C.

Because isoquercitrin increased the phosphorylation of AMPK and ACC, and reduced the gene expression levels of SREBP-1 and FAS in H4IIE cells, we investigated the critical role of AMPK signaling on isoquercitrin-mediated hepatic fatty acid metabolism using an AMPK inhibitor, compound C. Pretreatment of compound C significantly decreased the AMPK phosphorylation stimulated by isoquercitrin (Figure 4A). In addition, compound C significantly attenuated the mRNA expression of FAS induced by isoquercitrin in H4IIE cells (Figure 4B). These results indicate that AMPK plays a crucial role in the effect of isoquercitrin in regulating lipid metabolism in H4IIE cells.

### Isoquercitrin upregulates AdipoR1 mRNA expression via AMPK in H4IIE cells

**Figure 5:**
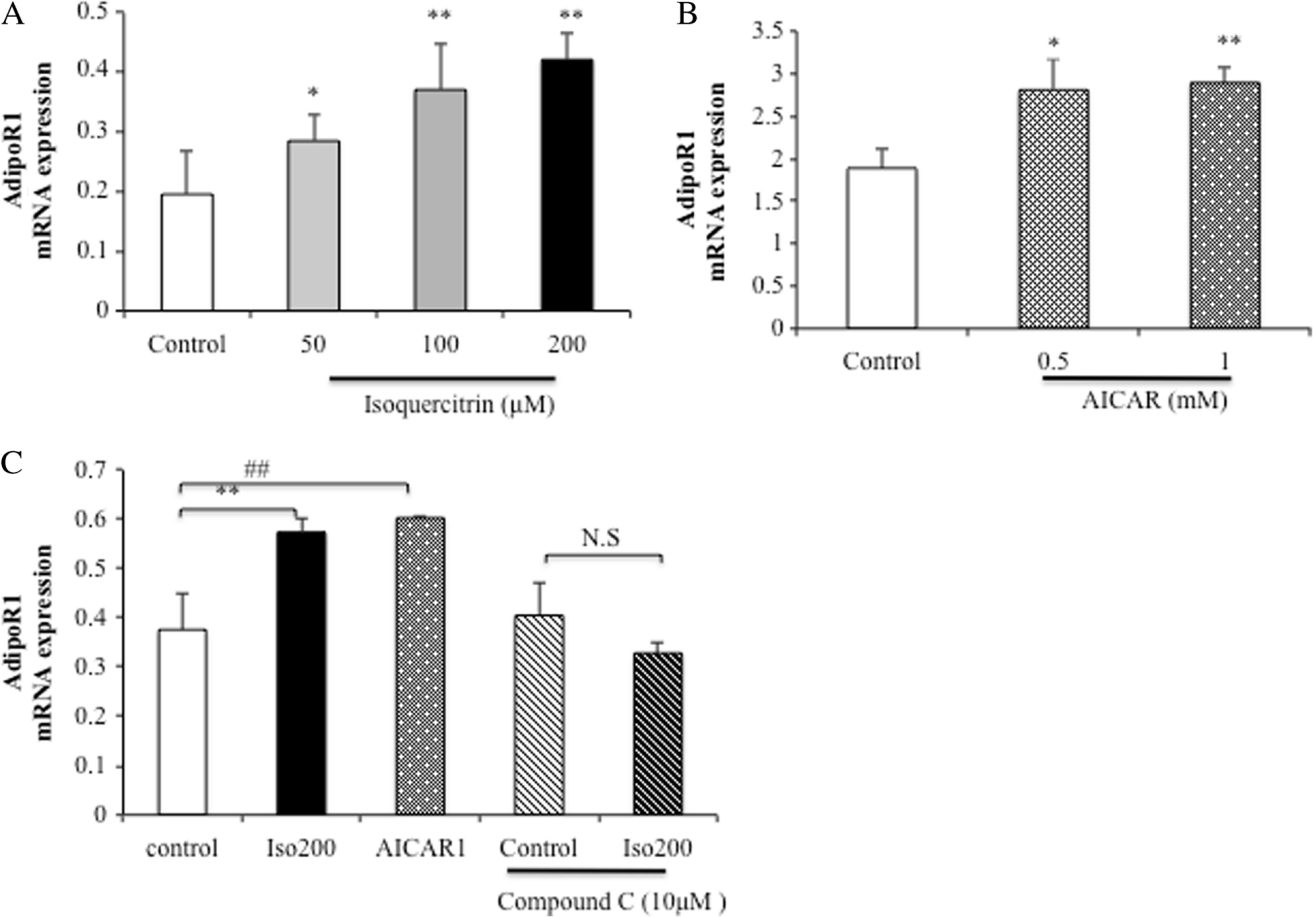
**Isoquercitrin increases AdipoR1 mRNA expression levels via AMPK in H4IIE cells. (A)** Cells were treated with different concentrations of isoquercitrin as mentioned. **(B)** H4IIE cells were treated with AICAR (0.5 mM and 1 mM) for 12 h as indicated. **(C)** H4IIE cells were treated with isoquercitrin (200 μM) or AICAR (1 mM) for 12 h in the absence or presence of preincubation with 10 μM compound C for 1 h. The mRNA expression levels of AdipoR1 were measured by quantitative real-time PCR and normalized to β-actin mRNA expression. Values are shown as means ± SEM (n = 3). **(A)** *P < 0.05; **P < 0.01 compared to control group. **(B)** *P < 0.05; **P < 0.01 compared to control group. **(C)** **P < 0.01 between isoquercitrin and control group, ##*P* < 0.01 between AICAR and control group, N.S. between isoquercitrin and isoquercitrin plus compound C.

Next, we studied whether isoquercitrin could regulate AdipoR1 mRNA expression levels in H4IIE cells by using real-time PCR. As expected, AdipoR1 mRNA expression in H4IIE cells was dose-dependently increased by isoquercitrin treatment (Figure 5A). Because isoquercitrin affects both the AdipoR1 mRNA expression level and AMPK phosphorylation, we investigated whether isoquercitrin stimulates the expression of AdipoR1 to result in AMPK activation, or if phosphorylating AMPK leads to an increase in AdipoR1 expression. First, we examined the effect of AMPK phosphorylation mediated by isoquercitrin on AdipoR1 mRNA expression levels by using the AMPK agonist AICAR and the AMPK inhibitor compound C. The results showed that the mRNA expression level of AdipoR1 was significantly increased by AICAR treatment in the H4IIE cells (Figure 5B). Pretreatment of H4IIE cells with compound C significantly suppressed the isoquercitrin-induced increase in AdipoR1 mRNA expression compared with AICAR treatment, which acted as a positive control (Figure 5C). These findings indicated that isoquercitrin might regulate the AdipoR1 mRNA expression level via AMPK.

### Isoquercitrin affects AMPK signaling after AdipoR1 knockdown in H4IIE cells

**Figure 6:**
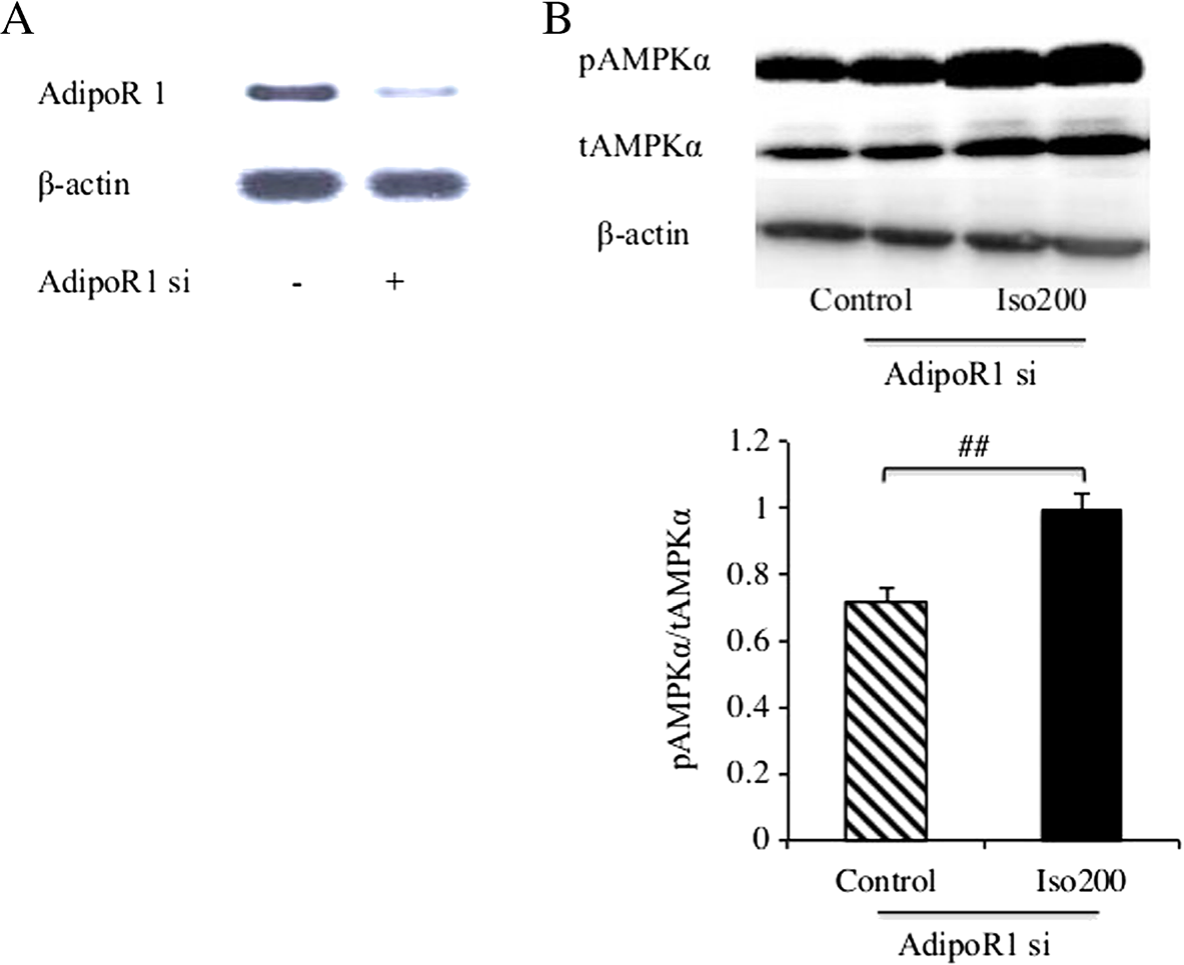
**AdipoR1 knockdown does not change isoquercitrin-induced AMPK signaling in H4IIE cells.** H4IIE cells were transfected with AdipoR1 siRNA or control siRNA as indicated, followed by isoquercitrin treatment for 12 h. **(A)** After transfection, AdipoR1 expression was determined by quantitative RT-PCR. **(B)** Phosphorylation and total AMPKα protein expression were measured by Western blotting. Results were analyzed and shown as means ± SEM (n = 3). #*P* < 0.05 between isoquercitrin and control plus AdipoR1 siRNA.

To evaluate whether isoquercitrin enhances AMPK phosphorylation without regulating AdipoR1, H4IIE cells were transfected with scrambled siRNAs, which served as the control, or with specific siRNAs against AdipoR1. After transfection, AdipoR1 mRNA expression in H4IIE cells was remarkably reduced when compared with the control, suggesting that the knockdown was effective (Figure 6A). The transfection of AdipoR1 siRNA in H4IIE cells did not alter AMPK activation stimulated by isoquercitrin treatment compared with the control group (Figure 6B).

## Discussion

AMPK is a master regulator and a major cellular energy sensor of metabolic homeostasis. Defects in AMPK are associated with metabolic disorders. The increase in the prevalence of lipid metabolic disorders has become a major global public health problem and plays a large role in the subsequent development of NAFLD. Therefore, there has been a worldwide focus on developing prevention methods and therapeutics that target lipid metabolic disorder and NAFLD. Recently, researchers have focused on the clinical effects of dietary compounds from plants, which are known to possess biological properties that reduce the risk of metabolic disorders. Previous *in vivo* and *in vitro* studies have suggested that several flavonoids, such as tiliroside, luteolin, and cathechins, act to manage metabolic disorders. Some of the main effects of these dietary compounds are on hepatic lipid metabolism via AMPK activation and FAS gene expression [13,16,17]. In the present study, we demonstrate that isoquercitrin, a quercetin glucoside, regulates the AMPK pathway to inhibit lipid accumulation in H4IIE cells. Thus, isoquercitrin may be a novel dietary flavonoid that can suppress the development of lipid metabolic disorder and NAFLD.

Numerous studies have indicated that AMPK is a key cellular energy sensor, which plays an important role in regulating the liver and whole-body lipid metabolic homeostasis. In many reports, it has been proved that the extent of phosphorylation of Thr172 reflects the degree of AMPK activation, which is necessary and sufficient for AMPK activity [18,19]. The first downstream enzymatic target of AMPK is ACC, which is related to the synthesis of malonyl-CoA. ACC is involved with promoting fatty acid biosynthesis and inhibiting fatty acid oxidation. The suppression of ACC activation by AMPK phosphorylation causes a rate-controlling decrease in malonyl-CoA, followed by a decrease in fatty acid synthesis and an increase in fatty acid oxidation. It also has been reported that phosphorylation of ACC at Ser77 and Ser79 participate in the control of ACC activity [20]. The present study showed that isoquercitrin treatment significantly stimulated AMPK activation and increased the phosphorylation of ACC.

SREBP-1c is a transcription factor, which stimulates fatty acid biosynthesis in the liver by regulating several lipogenic enzymes, such as FAS and ACC. In addition, it has been reported that the expression of SREBP-1 is markedly altered in the liver of obese patients and in the fatty livers of obese mice [2,21]. The action of SREBP-1 is suppressed by AMPK activation and mediates inhibition of ACC and FAS gene expression to regulate liver lipid metabolism in animal models [1]. In addition, some insulin-sensitizing drugs activate AMPK and subsequently, reduce hepatic levels of SREBP-1 and lipogenic gene expression in hepatocytes [3].

Consistent with these reports, our results indicated that isoquercitrin treatment induced a reduction in SREBP-1 mRNA expression levels and a downregulation of its target lipogenic gene FAS in H4IIE cells. Furthermore, pretreatment with compound C reversed the isoquercitrin-mediated decrease in FAS mRNA expression and inhibition of lipid deposition. These observations implied that the enhancement of AMPK activation by isoquercitrin leads to suppression of SREBP-1 and FAS gene expressions, resulting in the isoquercitrin-induced reduction in fatty acid accumulation in H4IIE cells.

AdipoR1 and AdipoR2, two major physiological receptors for adiponectin, have been reported to play critical roles in lipid and glucose metabolism [22]. Increasing the expression levels of AdipoR1 and AdipoR2 in the liver of mouse models of obesity and type 2 diabetes can improve insulin resistance and diabetes. Moreover, the disruption of AdipoR1 and AdipoR2 almost completely abolished adiponectin binding in the liver, leading to an increase in triglyceride content, oxidative stress, and inflammation, which resulted in NAFLD and insulin resistance. In addition, it has been reported that the expression levels of AdipoR1 and AdipoR2 are significantly decreased in NAFLD animal models [23]. A recent report showed that disruption of AdipoR1 and AdipoR2 in mice caused the mice to be more vulnerable to insulin resistance than adiponectin-knockout mice. This result indicates that the expressions of AdipoR1 and AdipoR2 may be regulated by more than adiponectin binding and adiponectin actions [22]. AdipoR1 has been found to be involved in activating the AMPK pathway, which mediates lipid metabolism in the liver and regulates globular adiponectin-stimulated AMPK activation in hepatocytes [24]. These studies suggest that increasing AdipoR1 will be a novel prevention approach for metabolism disorders.

Previous studies reported that AdipoR expression is regulated by hormones, cytokines, and metabolism factors, and it is also modified by adiponectin, FFA, the liver X receptor, and Peroxisome proliferator-activated receptor alpha (PPAR-α) and peroxisome proliferator-activated receptor gamma (PPAR-γ) agonists [25-27]. Recently, several flavonoids have been reported to regulate the expression of AdipoR1 and AMPK [28,29]. However, it was unknown whether isoquercitrin affects hepatic AdipoR1 mRNA expression and whether AMPK mediates fatty acid metabolism. In this study, we demonstrate that isoquercitrin upregulated the mRNA expression level of AdipoR1 and stimulated AMPK phosphorylation to inhibit lipid accumulation in H4IIE cells.

Many studies have shown that AdipoR1 is tightly related to activating downstream steps in the AMPK pathway to inhibit hepatic fatty acid synthesis and increase fatty acid oxidation. Interestingly, the results of the present study suggest that AMPK activation also increases the mRNA expression level of AdipoR1. The AMPK inhibitor decreased isoquercitrin-induced FAS expression and lipid deposition in H4IIE cells, implying that isoquercitrin might be a potent AMPK activator. Meanwhile, isoquercitrin increased AdipoR1 expression without adiponectin stimulation, whereas pretreatment with the AMPK inhibitor attenuated the isoquercitrin-mediated increase in AdipoR1 expression levels. In addition, the AMPK agonist AICAR significantly stimulated the mRNA expression level of AdipoR1. Therefore, it seems that isoquercitrin acts as an AMPK activator affecting the expression of AdipoR1, which is consistent with the stimulatory effect of the PPARα agonist on the regulation of AdipoR2 expression in liver cells [30,31].

Furthermore, our results demonstrate that isoquercitrin activated AMPK compared with the control group in an experiment using transfection of AdipoR1 siRNA in H4IIE cells, which indicates that the main effect of isoquercitrin is on AMPK activation. Thus, by acting as AMPK activator, isoquercitrin may regulate AMPK activation, leading to improve AdipoR1 expression in H4IIE cells. These data show, for what we believe is the first time, that AMPK agonists may increase the mRNA expression level of AdipoR1. This result requires further confirmation in the future.

## Conclusions

The present study indicates the novel finding that isoquercitrin, a dietary flavonoid in herbs, flowers, and vegetables, activates the AMPK signaling pathway in H4IIE cells. Isoquercitrin appeared to stimulate AMPK activation, which enhanced AdipoR1 expression, suppressed SREBP-1 and FAS expressions in H4IIE cells, and resulted in the regulation of fatty acid metabolism. These findings provide cellular molecular evidence demonstrating that isoquercitrin can potentially be used as a novel dietary compound to prevent lipid metabolism disorder and NAFLD. These results need to be investigated in further *in vivo* studies.

## Abbreviations

ACC: Acetyl-CoA carboxylase; AdipoR1: Adiponectin receptor 1; AdipoRs: Adiponectin receptors; AMPK: AMP-activated protein kinase; DMEM: Dulbecco’s Modified Eagle Medium; DMSO: Dimethyl sulfoxide; FAS: Fatty acid synthase; FFAs: Free fatty acids; NAFLD: Nonalcoholic fatty liver disease; PPAR-α: Peroxisome proliferator-activated receptor alpha; PPAR-γ: Peroxisome proliferator-activated receptor gamma; siRNA: Small interfering RNA; SREBP-1: Sterol regulatory element binding protein transcription factor 1.

## Competing interests

The authors have declared no financial or commercial competing interest.

## Authors’ contributions

JZ designed the study, conducted the experiments, and wrote the manuscript. HY performed the experiments, analyzed the data. GM designed the study and wrote the manuscript. WS, LQ, and LW performed the experiments and manuscript preparation. BZ, LH analyzed the data. XG drafted the manuscript. TL provided the initial idea, instructed the study. All authors read and approved of the final manuscript.

## Pre-publication history

The pre-publication history for this paper can be accessed here:

http://www.biomedcentral.com/1472-6882/14/42/prepub
